# Mutations in Non-Acid Patch Residues Disrupt H2A.Z’s Association with Chromatin through Multiple Mechanisms

**DOI:** 10.1371/journal.pone.0076394

**Published:** 2013-10-01

**Authors:** Thomas J. Wood, Angela Thistlethwaite, Michael R. Harris, Simon C. Lovell, Catherine B. Millar

**Affiliations:** Faculty of Life Sciences, University of Manchester, Manchester, United Kingdom; Texas A&M University, United States of America

## Abstract

The incorporation of histone variants into nucleosomes is a critical mechanism for regulating essential DNA-templated processes and for establishing distinct chromatin architectures with specialised functions. H2A.Z is an evolutionarily conserved H2A variant that has diverse roles in transcriptional regulation, heterochromatin boundary definition, chromosome stability and DNA repair. The H2A.Z C-terminus diverges in sequence from canonical H2A and imparts unique functions to H2A.Z in the yeast *S. cerevisiae*. Although mediated in part through the acid patch-containing M6 region, many molecular determinants of this divergent structure-function relationship remain unclear. Here, by using an unbiased random mutagenesis screen of H2A.Z alleles, we identify point mutations in the C-terminus outside of the M6 region that disrupt the normal function of H2A.Z in response to cytotoxic stress. These functional defects correlate with reduced chromatin association, which we attribute to reduced physical stability within chromatin, but also to altered interactions with the SWR and INO80 chromatin remodeling complexes. Together with experimental data, computational modelling of these residue changes in the context of protein structure suggests the importance of C-terminal domain integrity and configuration for maintaining the level of H2A.Z in nucleosomes.

## Introduction

H2A.Z is a highly conserved variant of histone H2A that is essential for viability in many organisms. Although lack of H2A.Z, encoded by the *HTZ1* gene [1], is not lethal in *S. cerevisiae*, *htz1∆* cells are sensitive to a range of drugs, including those that cause DNA damage or destabilise microtubules [2-5]. At the molecular level, functions for Htz1 include preventing the spread of heterochromatin proteins into euchromatin [6]; regulation of inducible genes [2,7,8]; directing the relocalisation of a permanent double-strand break to the nuclear periphery [9]; and recruiting the SUN-domain protein Mps3 to the nucleus [10]. With the exception of Mps3 recruitment, these functions all rely on the association of Htz1 with nucleosomes, which is regulated by the SWR and INO80 chromatin remodeling complexes (SWR-C and INO80-C) [4,5,11,12]. The SWR-C is responsible for deposition of Htz1 into chromatin through an ATP-dependent reaction catalysed by the Swr1 subunit, which swaps canonical H2A for Htz1 [11]. The reverse reaction, ejecting Htz1 and replacing it with H2A, can be carried out by the INO80-C [12] and by SWR-C when nucleosomal H3 is acetylated at K56 [13]. Both complexes contain multiple subunits that are required for their histone exchange functions [14-17].

**Figure 1:**
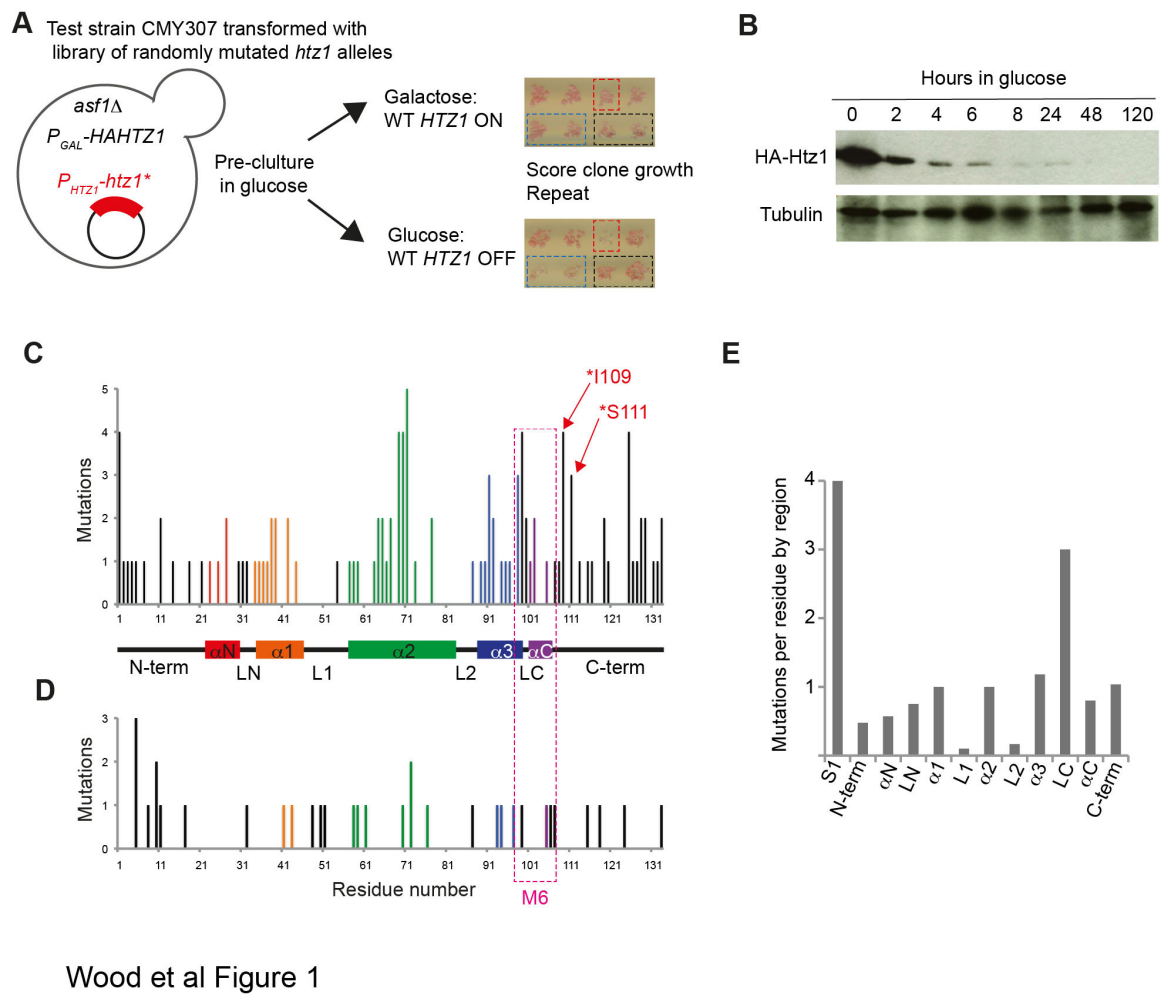
Random mutagenesis identifies residues in the unstructured regions of Htz1 that are required for function. **[A]** Overview of random mutagenesis screen. Yeast strain CMY307 containing *P*_*GAL1*_-regulated *HA-HTZ1* and *asf1∆* was transformed with a library of randomly mutated *htz1* alleles. Individual clones were picked into 96-well plates and pre-grown in glucose for 72H to repress *P*_*GAL1*_ *-HA-HTZ1* before plating in duplicate onto SG-Ura and SD-Ura to assess growth in the presence and absence of WT Htz1. CMY307 transformed with plasmids carrying WT Htz1 (black box) or empty vector (blue box) were included as controls on each plate. An example of a glucose-sensitive clone is shown boxed in red. **[B]** Western blot of protein lysates prepared from CMY307 grown in galactose and at various time-points after glucose addition show that WT HA-Htz1 protein is undetectable by 48H. **[C]** Frequency of mutations at each Htz1 residue recovered from non-functional alleles identified by screening as described in **[A]**. The coloured bars in the graph represent residues within known structured regions. A schematic of H2A.Z protein structure, where boxes depict α-helices, the black bars indicate unstructured regions and the M6 region is boxed in pink, is shown. The N- and C-terminal tails and the inter-helical loops (LN, L1, L2, LC) are also labelled and residues I109 and S111 are indicated with red arrows. **[D]** The frequency of mutations at each residue in a sample of clones from the random mutagenesis library that were not screened for Htz1 function in yeast. **[E]** Summary of mutation frequencies per residue in each structural region of Htz1. The protein sequence was divided into regions including the N- and C-termini, the various alpha helices and the inter-helical loops, as indicated in **[C]**. The mutation frequency for each region was normalised to the number of residues in that region to allow comparison. S1 (serine 1) was treated separately as most of the N-terminal mutations were at this residue.

Despite considerable sequence differences between H2A.Z and H2A – the proteins are about 40% different – the structure of nucleosome core particles (NCPs) containing H2A.Z are almost identical to canonical NCPs [18]. The numerous close contacts between the 8 histone subunits and between histones and DNA in NCPs mean that the central portion of each histone protein, the histone fold, is structurally critical. In H2A.Z, there are 3 central alpha helices (α1- α3) that form the histone fold domain and two additional alpha helical portions, αN and αC, that lie within the NCP (see Figure 1C for schematic). The N- and C-terminal tails of H2A.Z, which are upstream and downstream of the αN and αC helices respectively, extend outwards from the NCP and are unstructured.

The differences in amino acid sequence between H2A and H2A.Z are likely to underlie their different functions. Early domain-swap experiments that replaced short stretches of H2A.Z amino acids with H2A residues identified the “M6” region that includes the αC helix and flanking residues in the LC loop and the C-terminus as essential for H2A.Z function, as H2A.Z alleles carrying the corresponding amino acids from H2A failed to rescue viability defects in *Drosophila* [19]. In *S. cerevisiae*, the use of fusion proteins that joined the Htz1 N-terminus to the H2A C-terminus at a point within the M6 region also showed that the C-terminus of Htz1 is important for Htz1-specific functions in gene regulation and resistance to cytotoxic stress [2,3]. More recently, a molecular explanation for the importance of the M6 domain has been provided as Htz1 protein carrying the H2A M6 region does not interact with the SWR-C and is therefore not assembled into chromatin in the normal fashion [14]. Further to this, specific acidic residues within the M6 region are critical for Htz1 deposition [20]. Normal SWR-C-mediated assembly of Htz1 into nucleosomes is upstream of almost all other known Htz1 functions, explaining why the M6 region is integral to the functions of Htz1.

Outside of the M6 region, recent studies have shown that deleting portions of the N- and C-terminal tails alters the genetic interaction profiles for *htz1* alleles, indicating that the tails are functionally important [21,22]. Narrowing down from regions to individual amino acids, some residues of Htz1 are known to be important through acting as post-translational modification sites. Four lysines in the N-terminal tail of Htz1 are acetylated and these sites are important for restricting heterochromatin spread, inducible gene expression and normal chromosome segregation [23-27]. There are also post-translationally modified sites in the C-terminus, where K125 and K132 can be sumoylated, and these residues are important for relocalisation of a persistent double strand break to the nuclear periphery [9]. The C-terminus also contains a triplet of residues (118-120) that are collectively required for Htz1 functions [22]. Systematic mutation of all Htz1 residues to alanines has identified 7 residues scattered across the protein that contribute to resistance to various drugs [28]. As the molecular reasons for drug sensitivity phenotypes in *htz1∆* cells are complicated, probably involving altered SWR-C activity [26,29], it is likely that other residues in Htz1 are functionally important but that their loss may not cause drug sensitivities. To test this possibility, we have capitalized on the synthetic growth defect of an *htz1∆asf1∆* strain as a read-out for loss of Htz1 function. Asf1 is a histone chaperone and *asf1∆* cells have defects in many of the same processes as *htz1∆*strains, including silencing, gene regulation and the DNA damage response [30-33]. We therefore hoped to identify novel functionally important residues in Htz1 that might be important for any or all of these pathways. Here we present the results of this screen, which show that this screening read-out indeed identifies novel functionally important residues in Htz1. Additionally, we identify roles for two single residues outside of the M6 domain, I109 and S111, in regulating Htz1’s association with chromatin.

## Results

### Mutations that impair Htz1 function cluster in the C-terminus of the protein

In order to identify novel determinants of Htz1 function outside of the M6 region, we took advantage of the fact that Htz1 is required for viability in the absence of the histone chaperone Asf1 [34]. A strain carrying a glucose-repressible HA-tagged *HTZ1* allele and deleted for *ASF1* was used to identify mutant *htz1* alleles generated by error-prone PCR that could not provide the wild-type function of Htz1 (see Figure 1A for overview of the screen). When grown in glucose-containing medium, WT Htz1 protein is not produced (Figure 1B), and the *asf1∆* cells grow poorly or not at all. 1453 randomly mutated *htz1* alleles were tested for their ability to rescue growth of this strain on glucose. After two rounds of screening, 149 mutant alleles that failed to rescue growth were isolated. These clones were sequenced to identify the mutations causing loss of function. 60% of the recovered mutant alleles encoded a protein truncated before the end of the α-helical portion of the protein, including 10 clones that harboured mutations in the initiator methionine. All of these truncated clones were discounted from further analysis.

59 clones with point mutations or those truncated after the structured portion of Htz1 were selected for further analysis. Although sequencing of cloned error-prone PCR products had initially shown that the clones contained an average of one mutation per coding sequence, many of the clones that were recovered after screening in yeast contained more than one mutation. This is probably due to the fact that we selected only very growth defective clones for sequencing. To identify functionally important residues, we therefore examined the frequency of mutation at each residue from the 59 clones. The average mutation rate per residue in this dataset is 0.86. Visual inspection of the mutation frequency at each residue when mapped onto the known structural domains of Htz1 revealed that mutations within α-helices, particularly in the centre of the longest helix, α2 are poorly tolerated, as are mutations in the C-terminus (Figure 1C). To ensure that this pattern is not due to bias within the library, clones that had not been selected for loss of Htz1 function (i.e. plasmids that had not been transformed into yeast) were also sequenced and these show a more equal distribution of mutations across the protein sequence (Figure 1D). To assess which regions of Htz1 had a higher than expected mutation frequency, the protein was divided into regions based on the secondary structure and the number of mutations in the selected clones falling in each region was normalised to the number of residues (Figure 1E). This analysis revealed that the LC loop between the α3 and αC helices, which is in the centre of the M6 region, had the highest relative number of mutations and that mutations in the α-helices were recovered more frequently than mutations in the loops LN, L1, and L2. Unexpectedly, the C-terminus of the protein had a mutation frequency comparable to the α-helices.

Mutations that fell within α-helices of the histone fold domain, or within the M6 region, were expected to disrupt protein structure or interaction with the SWR-C deposition complex, respectively, and therefore were not examined further. At the individual residue level, all of the remaining residues that showed a high mutation frequency, with the exception of S1 and S11 at the N-terminus, were located in the Htz1 C-terminus, including I109, S111, N119, and 4 of the 6 C-terminal lysines. The C-terminal lysines of Htz1 have previously been implicated in the DNA damage response [9] and N119 has been implicated in Htz1’s chromatin association [22] but I109 and S111 (indicated on Figure 1C) were of particular interest because they occurred at positions within the C-terminal docking domain but outside of the M6 region, and had not been characterised previously at the molecular level. Some mutations in the C-terminal region were nonsense mutations resulting in truncated proteins and analysis of these revealed a role for the Htz1 C-terminus in stabilising the Htz1 interaction with nucleosomes [35]. However, such mutations are relatively crude, removing between 6 and 23 residues from the C-terminus of the protein, and we hypothesised that single residue changes might reveal nuances in C-terminal function that would not be detectable if the entire domain was absent. We therefore proceeded to examine the effects of the I109T and S111P point mutations on Htz1 function in comparison to a mutant that truncates Htz1 at position 110 (∆*111-133*). Mutant plasmid constructs were re-generated to ensure that they contained only the mutation in question before use in subsequent analyses.

### Docking domain point mutations outside of the M6 region, I109T and S111P, abrogate the function and chromatin association of Htz1

**Figure 2:**
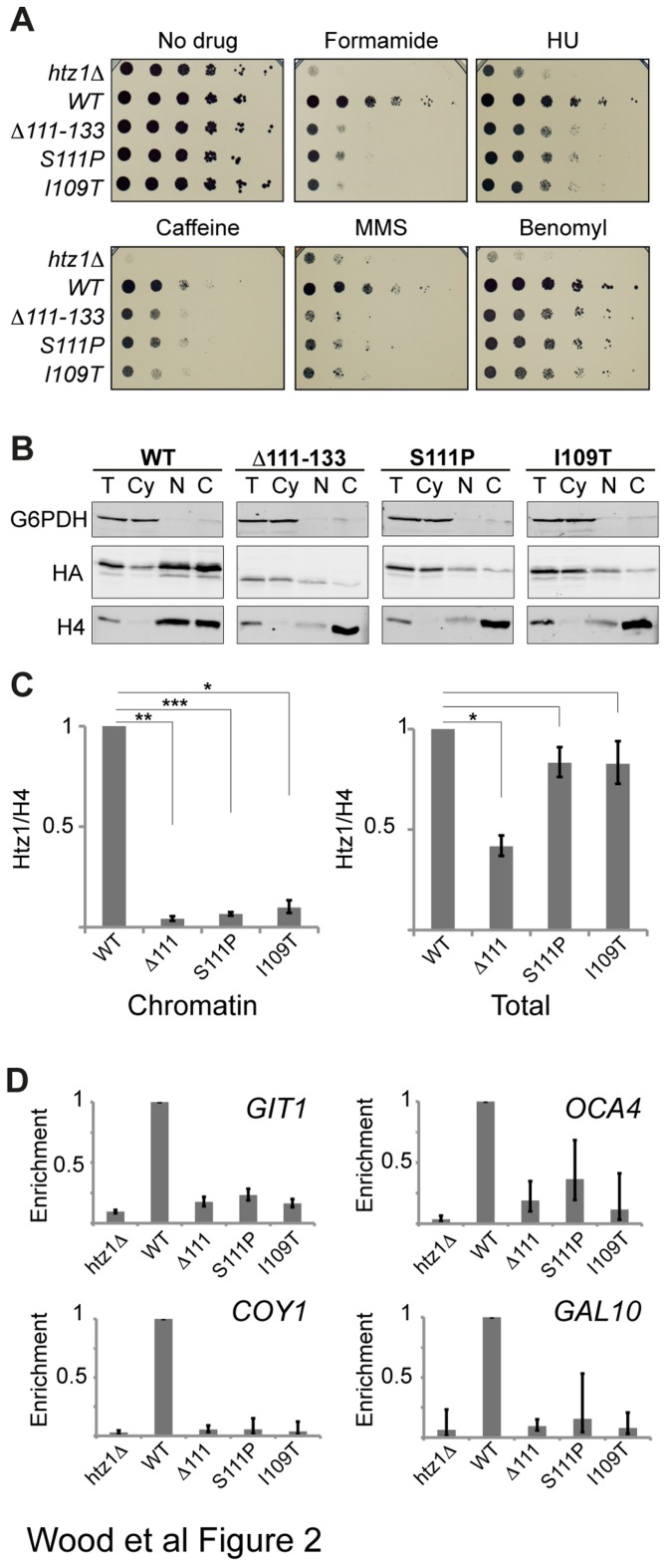
I109T and S111P point mutations disrupt normal Htz1 function and chromatin association. **[A]** Htz1 C-terminal mutants are sensitive to cytotoxic stress. To compare the growth of strains carrying WT or mutant *htz1* alleles in the presence of the indicated drugs, cells were serially diluted 1:5 and spotted onto plates, from left to right in each panel. The identities of the *htz1* alleles are indicated at the left of the panel. **[B]** C-terminal mutants have a chromatin association defect. Sub-cellular fractionation was used to isolate cytosolic (Cy), nuclear (N) and insoluble chromatin fractions (C), from total cell lysate (T). Fractions were analysed by SDS-PAGE and immunoblotting, with anti-G6PDH used as a cytoplasmic loading control, anti-histone H4 as a chromatin loading control, and anti-HA used to detect HA-tagged Htz1. The identity of the Htz1 protein is indicated above each panel. **[C]** Quantification of HA-Htz1 protein levels normalised to H4 (Htz1/H4) from Western blots. Graphs show the averages of mutant HA-Htz1 levels normalised to the WT level in chromatin (left) and total protein (right) fractions from four independent experiments. Error bars correspond to the standard error of the mean and asterisks indicate the results of two-tailed paired *t*-tests between WT and the corresponding mutant, where * = *P* < 0.01, ** = *P* < 0.005, *** = *P* < 0.001. **[D]** ChIP analysis of WT and mutant Htz1 proteins reveals reduced mutant Htz1 occupancy. Htz1 enrichment at the heterochromatin (HMR) flanking genes *GIT1* and *OCA4* and the euchromatic genes *COY1* and *GAL10* were calculated relative to a negative control region within the silent mating type locus *HMR*. Average mutant enrichments are shown relative to WT; error bars represent standard deviations from two replicates.

To further assess the functional consequences of I109T and S111P mutations on Htz1 function, we investigated whether strains expressing these mutated proteins would have other phenotypes corresponding to Htz1 loss-of-function. I109T and S111P mutations, like *htz1∆*, reduced the resistance of cells to a range of drugs (Figure 2A). Mutants exhibited intermediate sensitivity to formamide, hydroxyurea and caffeine, but were only mildly affected in response to benomyl, and were as sensitive as *htz1∆* on MMS. In each case, the truncation and point mutants behaved similarly, suggesting that in general the point mutations do not affect Htz1 function in ways that are distinct from the effects of deleting the entire region.

Given that Htz1 function is generally dependent upon the incorporation of Htz1 into chromatin, we wanted to investigate whether the loss-of-function observed in the docking domain point mutants could be attributed to a defect in chromatin association. Indeed, when sub-cellular biochemical fractionation and immunoblotting were performed under normal growth conditions, the amount of point mutated Htz1 in the chromatin pellet was reduced significantly (paired *t*-test, n = 4 (biological replicates), *P* < 0.005, Figure 2B, C), by more than 10-fold, despite the total level of cellular protein being close to WT levels in the point mutants and only 2.4-fold lower in the truncation mutant (Figure 2C, right). Chromatin immunoprecipitation experiments verified this depletion from chromatin at a number of Htz1-enriched loci (Figure 2D). We next explored the possible causes of the observed depletion of Htz1 mutant proteins from chromatin. The steady-state level of Htz1 associated with chromatin is the combined result of opposing activities that incorporate or remove Htz1 from chromatin and we therefore perturbed both deposition and removal pathways to assess their effects.

### I109T and S111P mutants have diminished interaction with the SWR deposition complex

As the SWR-C is responsible for catalysing the deposition of Htz1 into nucleosomes [11], we examined whether the reduced level of mutant Htz1 proteins in chromatin could be the result of a defect in normal SWR-C-mediated deposition. We first tested whether the Htz1 mutants could interact with the nuclear chaperone Chz1 that delivers Htz1 to the SWR-C [36]. Co-immunoprecipitation experiments revealed that all the mutant forms of Htz1 could interact with Chz1 (Figure S1), in line with structural studies that show that Chz1 predominantly contacts Htz1 through the α1 and α2 helices and the intervening L1 loop that are towards the N-terminus of the protein [37].

**Figure 3:**
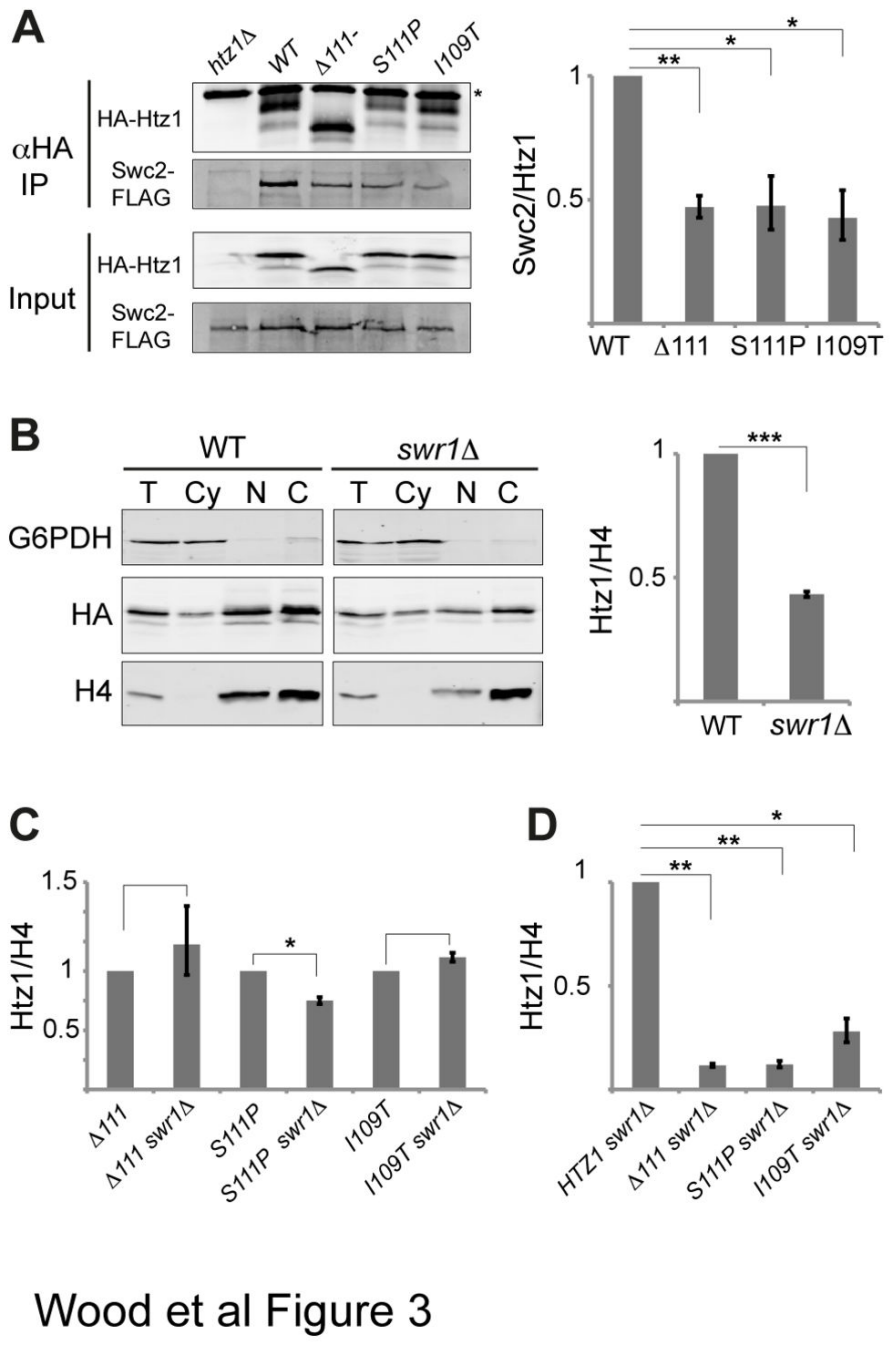
C-terminal mutants have reduced affinity for the SWR deposition complex. **[A]** Anti-HA antibodies were used to immunoprecipitate cell lysates from strains expressing FLAG-tagged Swc2 and either HA-tagged WT, mutant, or no Htz1 (htz1∆). Input and anti-HA IP samples were analysed by anti-HA and anti-flag immunoblotting, with an example blot shown on the left. The position of the antibody light chain is indicated (*). Levels of co-immunoprecipitated Swc2-FLAG for each strain were normalised to the amount of immunoprecipitated HA-Htz1, expressed relative to WT, and averages are depicted in the graph (right; n= 4). **[B]-[D]** Effects of deleting Swr1 on HA-Htz1 levels in chromatin. **[B]** Sub-cellular fractionation performed and labelled as described in Figure 2B for WT Htz1 in *SWR1* and *swr1∆* backgrounds. A representative blot is shown on the left, and chromatin levels of HA-Htz1 normalised to H4, expressed relative to WT and averaged are shown on the right (n = 3). **[C]** Quantification of chromatin HA-Htz1 protein levels as in **[B]** but where each double mutant is normalised to the corresponding single HA-Htz1 mutant (n = 3). **[D]** Quantification of chromatin HA-Htz1 protein levels as in **[B]** but where each double mutant is normalised to the WT *swr1∆* level (n = 3). Error bars indicate standard error of the mean. Asterisks indicate the results of two-tailed paired *t*-tests between the indicated strains, where * = *P* < 0.05, ** = *P* < 0.005, *** = *P* < 0.001.

The association of Htz1 with the SWR-C requires both the M6 region of Htz1 and the Swc2 subunit of the complex [14]. As I109 and S111 are close to the M6 region (Figure 1C), we investigated whether these mutations could disrupt interaction with the complex and therefore impair SWR-C-dependent deposition. HA-tagged Htz1 mutants were immunoprecipitated from cell lysates and immunoblotting was used to detect co-purifying Flag-tagged Swc2 (Figure 3A). Swc2 co-immunoprecipitated with all of the mutant proteins. However, normalisation of the amount of co-purified Swc2 to immunoprecipitated Htz1 (Figure 3A, right) indicated that the mutants had diminished *in vivo* interactions with SWR-C, relative to WT Htz1. We confirmed this finding with a second SWR-C subunit, Arp6, which does not directly contact Htz1 (Figure S2).

If Swr1-mediated Htz1 deposition is impaired in the Htz1 mutants, removal of Swr1 would not be expected to further lower the levels of the mutants in the chromatin fraction. Deletion of *SWR1* reduced the level of WT Htz1 in the chromatin pellet by an average of 2.3-fold (Figure 3B) and indeed the effect of deleting *SWR1* on mutant Htz1 chromatin levels was less pronounced. The reduction in chromatin levels was only 1.3-fold in the case of *S111Pswr1∆*, and was modestly increased (without statistical significance) in the *I109Tswr1∆* and *∆111swr1∆* mutants respectively (Figure 3C). These data suggest that normal SWR-C-mediated deposition of mutant Htz1 proteins is compromised by their reduced affinity for the SWR-C. However, C-terminal mutants in the absence of Swr1 still had a severe chromatin association defect relative to WT Htz1 in the absence of Swr1 (Figure 3D). This indicates that loss of SWR-C-mediated deposition cannot fully account for the reduced abundance of these mutants in chromatin.

### The INO80 complex contributes to the reduced levels of mutant Htz1 in chromatin

**Figure 4:**
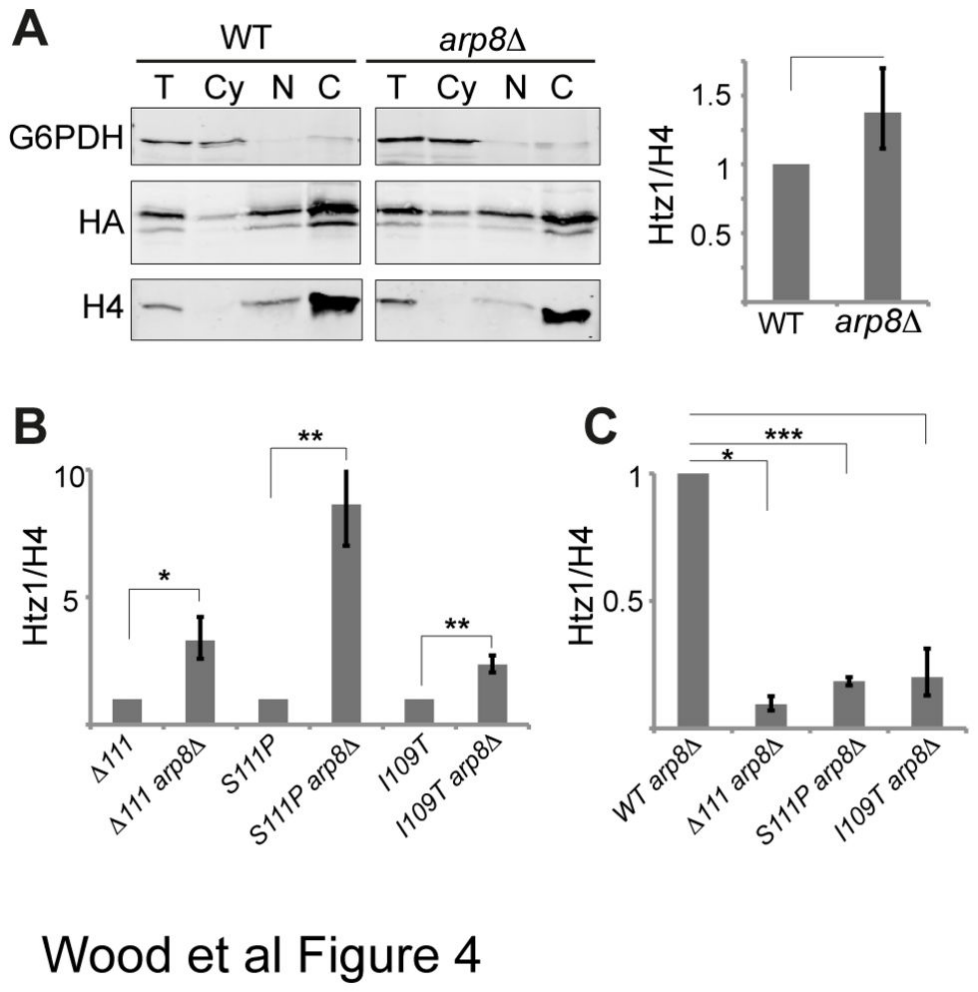
Loss of INO80 complex activity increases the association of C-terminal mutants with chromatin. **[A]** Western blots of sub-cellular fractions generated from WT and *arp8∆*cells, labelled as in Figure 2B. A representative example is shown and chromatin HA-Htz1 protein levels normalised to H4, expressed relative to WT and averaged from 3 biological replicates, are shown on the right. **[B]** Quantification of chromatin HA-Htz1 protein levels as in **[A]** but where each double mutant is compared to the corresponding single HA-Htz1 mutant (n = 3 for bars 1-4 from the left and n = 4 for bars 5 & 6). **[C]** Quantification of chromatin HA-Htz1 protein levels as in **[A]** but where each double mutant is normalised to the WT *arp8∆* level (n = 3). Error bars indicate standard error of the mean. Asterisks indicate the results of two-tailed paired *t*-tests between the indicated strains, where * = *P* < 0.05, ** = *P* < 0.01, *** = *P* < 0.005.

A decreased level of Htz1 mutant protein in chromatin could also be due to increased or constitutive removal by the INO80-C and we therefore tested whether inactivation of the INO80-C could rescue the abundance of mutant Htz1 proteins in the chromatin fraction. Deletion of *ARP8*, a subunit required for INO80 chromatin remodeling activity [17] modestly increased the chromatin level of Htz1 (1.4-fold) but without statistical significance (Figure 4A). Interestingly however, deletion of *ARP8* caused a 2.4-8.6-fold increase in the amount of mutant Htz1 in chromatin (Figure 4B). Similarly, in the absence of Arp5, which is also required for INO80 activity [17], we observed an increase in the association of mutant Htz1 with chromatin but not wild-type Htz1 (Figure S3). These experiments suggested that the INO80-C contributes more to regulating the mutant Htz1 protein chromatin level than the wild-type level. Although increased INO80-dependent eviction of mutant Htz1 provided an alternative explanation for the chromatin association defect, it did not exclude a simultaneous impairment of SWR-C deposition or some other mechanism. Indeed, even in the absence of Arp8, C-terminal mutants were still severely defective in chromatin association relative to WT Htz1 in the absence of Arp8 (Figure 4C). Therefore we next considered whether C-terminal point mutations could be affecting the stable retention of Htz1 in chromatin, independently of both upstream SWR-C-mediated deposition and downstream INO80-C-mediated eviction.

### Docking domain point mutations disrupt the physical stability of the association of Htz1 with chromatin

**Figure 5:**
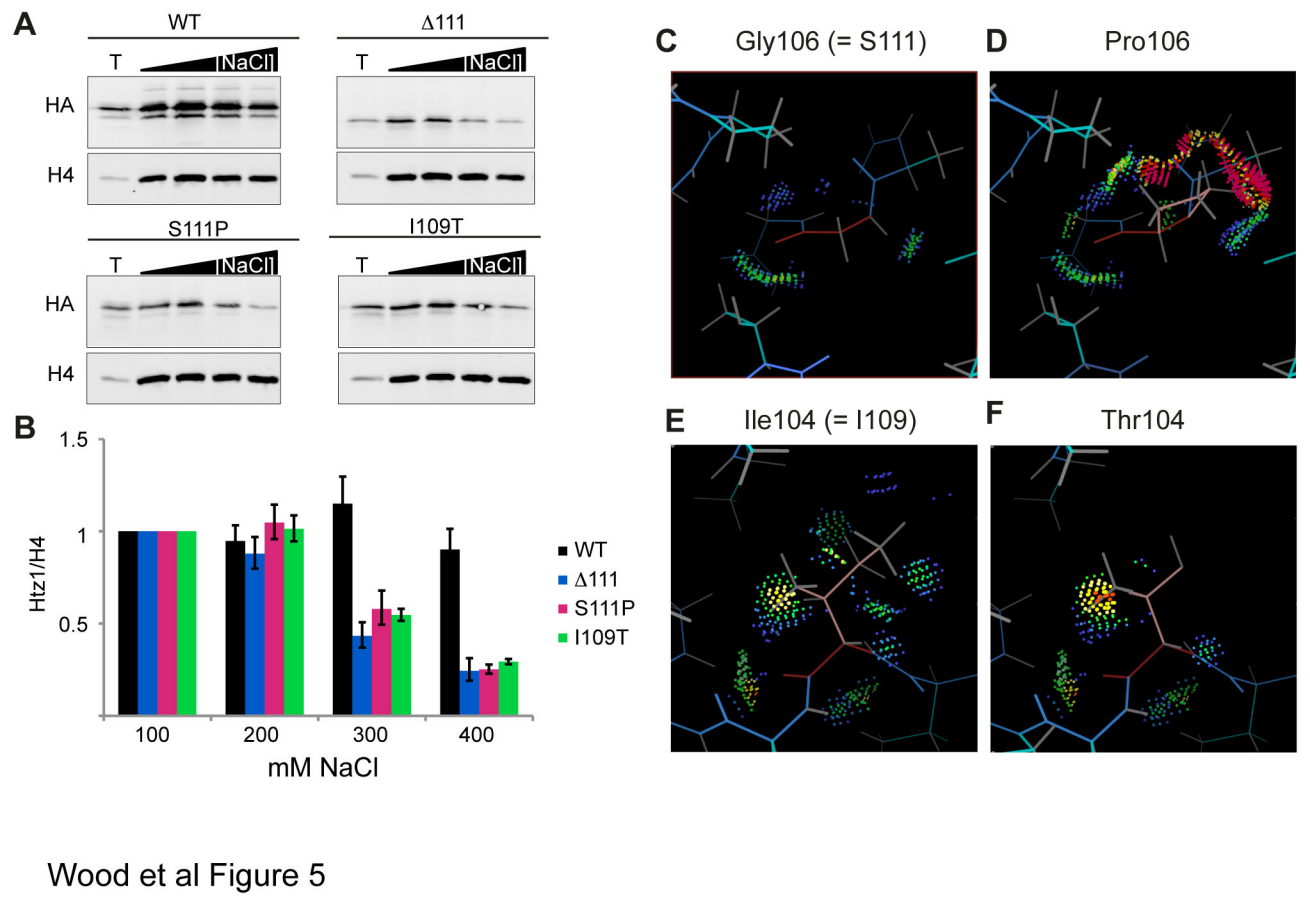
Point mutations in the nucleosome docking domain reduce the physical stability of Htz1’s association with chromatin. **[A]-[B]** Mutants are less resistant to washing with increased ionic-strength buffers. **[A]** HA-Htz1 levels in total cell lysates (T), and in insoluble chromatin fractions after washing with buffers containing 100, 200, 300 or 400 mM NaCl were determined by immunoblotting with anti-HA and anti-H4. **[B]** For each strain, levels of HA-Htz1 normalised to H4 for each washed chromatin sample were compared to the 100 mM wash sample and averaged (n = 3). Error bars indicate standard error of the mean. **[C]-[F]** Computational modelling of point mutations at equivalent positions in the mouse H2A.Z crystal structure predicts changes in local intermolecular interactions. KiNG software was used to model the effect of mutating residues in the mouse H2A.Z nucleosome crystal structure [18], at the positions equivalent to yeast serine 111 and isoleucine 109 (glycine 106 and isoleucine 104 respectively). In each panel, blue, grey and cyan lines represent the main chains, hydrogen atoms and side chains respectively, apart from G106, I104 and the residues substituted at these sites, where the main chains are coloured red and the side chains are pale pink. Green and blue dots indicate stabilising van der Waals interactions; yellow, orange and red spikes indicate small intermolecular clashes and pink spikes indicate large clashes. Local interactions are modelled for G106 **[C]**, but when substituted with proline **[D]** substantial van der Waals overlaps are seen. I104 makes several stabilising van der Waal’s contacts **[E]**, which are lost upon substitution with threonine **[F]** (right hand side of panel). In addition the hydrogen-bonding potential of the threonine hydroxyl group is unsatisfied.

The location of I109T and S111P mutations within the nucleosome docking domain of Htz1 suggested that the physical stability of Htz1 within the nucleosome might be directly affected. To investigate this possibility we performed the sub-cellular fractionation assay as before, but washed the chromatin pellet in solutions of increasing ionic-strength, and observed changes in the Htz1 chromatin level (normalised to histone H4), relative to the level at sub-physiological ionic strength (100 mM NaCl) (Figure 5A, B). The level of WT Htz1 in the chromatin pellet was not significantly affected by washing with buffers of increasing ionic-strength (Figure 5A, B). In contrast, the level of mutant Htz1 in chromatin reduced noticeably at 300 mM, and by 400 mM had decreased by around 3-fold. This shows that point mutated Htz1 can be dissociated from chromatin more easily *in vitro*, and indicates a defect in the stability of its association with chromatin.

In order to predict the structural effects of the point mutations on the mutated Htz1 proteins that lead to the observed stability defects, we used computational modelling. Models were based on the crystal structure of a NCP containing mouse H2A.Z [18]. In each case, the mutation was modelled in all rotamers assuming no changes to neighbouring main chain or side chain conformations. Substitution of proline for glycine at position 106, which corresponds to serine 111, is predicted to cause several large intermolecular clashes with neighbouring upstream residues in our conservative models (Figure 5C and D). Since large van der Waals overlaps are non-physiological [38] where they are observed in the models, the most likely interpretation is that the path of the downstream C-terminal tail is redirected in order to alleviate the steric clashes. In the case of the I109T mutation, only small van der Waals overlaps are predicted. However, we observe the loss of stabilising van der Waals interactions between the Cδ methyl group of the isoleucine and the α3 helix and that the hydroxyl group of the introduced threonine residue has unsatisfied hydrogen bonding potential (Figure 5E and F), and these changes may also drive local rearrangement. In both models the likelihood of the reorganisation of the C-terminal tail suggests an explanation for the similarity of the phenotypic consequences of the S111P and I109T mutations to the complete removal of the C-terminal tail in the ∆111-133 mutant.

## Discussion

We undertook a random mutagenesis screen to identify novel functional residues in the budding yeast H2A.Z ortholog, Htz1. As expected, the majority of the clones unable to provide wild-type Htz1 function encoded truncated proteins that removed part of the structured portion of the protein. The prevalence of these clones (60%) may indicate that we recovered primarily complete loss of function clones in our screen. Within the remaining 40% of clones, we found that the loop between the α3 and αC helices, LC, was the region of Htz1 that showed the highest frequency of mutations, even higher than any of the α-helical regions (Figure 1E). This loop forms part of the M6 region that is required for Htz1’s interaction with the SWR-C and therefore its normal incorporation into chromatin. The alpha helices were expected to have a high mutation rate because of their structural importance, and our data show that mutations in the central portion of the α3 helix are most disruptive (Figure 1C). The C-terminal tail of Htz1 had a mutation frequency similar to the α1, α2 and α3 helices, indicating that the Htz1 C-terminus is functionally important, a conclusion that has also been reached through analysis of engineered truncation mutants [22]. Somewhat surprisingly, the N-terminus of Htz1 was not enriched for frequently mutated residues apart from serine 1. Further studies of serine 1 are ongoing to ascertain the role of this residue in Htz1 biology. Although our findings may imply that the N-terminus does not contain functionally important residues, there are other explanations for lower recovery of mutations in this region. Reasons may include redundancy between N-terminal residues, as has been described for the N-terminal acetylation sites [21], which would mean that mutations at individual sites are not phenotypically detectable. Additionally, although we chose an *asf1∆* strain for screening because these mutants are defective in many of the same processes as *htz1∆*strains, mutations that affect Htz1 function in a pathway unaffected by *asf1∆*would not be recovered using our approach. Therefore, although we have been successful in identifying new important residues, it is likely that still more could be revealed using a different selection procedure. Despite this, our screen has identified most of the previously identified functionally important residues in Htz1 as well as some novel ones. A study in which all non-alanine residues within Htz1 were converted to alanines revealed 7 residues that are required for resistance to one or more drugs [28]. The three residues that gave only mild drug sensitivity phenotypes (F32, L81, and G113) when mutated to alanine were not mutated above the background rate in our screen. However, the remaining 4 residues (Y65, E69, D98 and I109) identified by Kawano et al [28] were also frequently mutated in our screen. Similarly, we found the residues within the M6 region identified by Jensen et al’s [20] random and targeted mutagenesis, E100 and D98, mutated at high frequency. We also recovered an above background mutation frequency for N119, which was shown by Wang et al [22] to cause mild drug sensitivity when mutated together with H117 and I118 to alanine.

We have focused in this study on two novel mutations that lie in the C-terminal tail of Htz1, downstream of the M6 region, at residues I109 and S111. The single point mutations I109T or S111P rendered *htz1∆*cells sensitive to formamide, hydroxyurea, caffeine, MMS and benomyl. Comparison to complete loss of Htz1 and to loss of the entire non-structured portion of the C-terminus (∆111) revealed that although the point mutants were sensitive to all the same agents as a complete deletion of *HTZ1*, they are less sensitive than the deletion to all agents with the exception of MMS, where *htz1∆*cells have similar growth to cells carrying mutated alleles. In the case of benomyl, the point mutants have only a marginal sensitivity compared to *htz1∆*which may indicate that these mutant proteins do not compromise chromosome segregation. We observed some very slight differences in growth between the I109, S111 or ∆111 mutants on different drug plates but in general mutating either I109 or S111 produces growth defects similar to complete deletion of the C-terminal tail.

The phenotypic defects observed in the I109T and S111P mutants can be explained by the reduced abundance of these proteins in chromatin; however the reasons for reduced chromatin occupancy are complex. Interestingly, although I109 and S111 are outside the previously characterised M6 region that is required for Htz1 to interact with the SWR-C, we found that mutations at these sites reduced the ability of mutant Htz1 to interact with the SWR-C. This contrasts with the findings of Wang et al [22], who showed that truncated Htz1 lacking residues 109 and 111 and tagged with FLAG at the C-terminus does not have a reduced interaction with Swc2 As we have used N-terminally HA-tagged Htz1 constructs, it is possible that the different positions of the tags on Htz1 in the two studies affect the requirement for Htz1 C-terminal residues in the interaction with the SWR-C. Notably, the mutants described here were still able to interact with SWR-C subunits to some extent, and residues I109 and S111 are therefore important, but not essential for this interaction.

Consistent with a defect in the SWR-C-mediated assembly pathway, levels of the mutant proteins in the chromatin fraction do not show the dramatic decrease seen for WT Htz1 when *SWR1* is deleted. In fact, the levels of I109T and ∆111 proteins increase slightly, which may indicate that Swr1 directly or indirectly acts to remove these proteins from chromatin. Interestingly, Swr1 has recently been shown to remove Htz1 from nucleosomes that are acetylated at H3K56 [13] and this activity may explain our findings. However, although the I109T and S111P mutations can impair the SWR-C-mediated deposition pathway, perhaps because of a reduced affinity of the SWR-C for the proteins, it is notable that mutant proteins have a much more severe defect in chromatin association than the WT protein either in the presence or the absence of Swr1. This means that there must be more than one reason for the reduced chromatin occupancies of the mutant proteins, although we expect that altered interaction with the SWR-C as a consequence of these mutations would act upstream of any other defects.

The rate at which Htz1 is removed from nucleosomes is also an important determinant of Htz1 levels in chromatin. Htz1 removal happens partially through non-specific mechanisms, for example during the nucleosome disruption that happens during gene transcription [8,39], as well as through specific mechanisms such as ATP-dependent Htz1 replacement with H2A catalysed by the INO80-C [12]. Disabling the INO80-C had a more dramatic effect on the chromatin levels of mutant Htz1 proteins than on WT Htz1, with noticeable differences between mutants, indicating that the INO80-C removal pathway acts on these mutants and may even preferentially remove them. It would have been interesting to investigate the interplay between INO80-C and SWR-C in the context of these mutants but unfortunately we were not able to generate an *arp8∆swr1∆* double mutant in our strain background. As *ino80∆swr1∆* is also inviable [12], it seems that SWR-C and INO80-C act redundantly, perhaps through coordinated deposition and removal of Htz1, in an essential process. Interestingly, the level of the S111P mutant is increased substantially, by more than 8-fold, in the absence of INO80-C activity. It is not currently known how INO80-C recognizes Htz1-containing nucleosomes and it is possible that residue 111 influences this process. If this were the case, the role of S111 would appear to be in limiting interaction or activity, perhaps allosterically, as mutation of S111P led to greater depletion from chromatin by the INO80-C. However, although the removal of INO80-C activity can partially rescue the levels of mutant Htz1 in chromatin, the reduced chromatin abundance of these mutants can’t be fully explained by increased removal by INO80-C. In favour of a general destabilisation of the mutant proteins, we found that the resistance of the mutant proteins to washing with buffers of increasing ionic strength was lowered. This may be due to the location of I109 and S111 within the docking domain of Htz1 that provides an interaction interface with the histone H3-H4 tetramer [18]. Computational modelling revealed that mutations I109T or S111P would introduce molecular clashes that would need to be accommodated through local structural reconfiguration. At I109, the requirement for an isoleucine appears particularly stringent, as all canonical and variant H2A protein types have an isoleucine at this position. In line with this, we recovered mutations that changed I109 to asparagine or valine in addition to the I109T mutant that we have described in detail, and mutation of I109 to alanine has also been shown to affect Htz1’s functions [28]. Conversely we only identified mutations to proline at position 111 in this screen, which may mean that specific introduction of proline rather than lack of serine is disruptive. Only H2A.Z orthologs from closely related yeast species have a serine at this position; outside of these the most common amino acid at this position is glycine. S111 is located at the apex of a turn in the C-terminal tail and we hypothesise that the replacement of a small residue by a less flexible larger one may redirect the rest of the Htz1 C-terminus. Reconfiguration of the C-terminus due to loss of isoleucine at position 109 or to presence of proline at position 111 would explain why I109T, S111P and ∆111 have similar phenotypic effects.

Our findings place new emphasis on the importance of residues outside of the M6 region in H2A.Z function, and highlight the stringent requirement for structural integrity at these positions. Changes to the protein sequence in this region can disrupt Htz1 chromatin abundance at several levels: by impairing interaction with the deposition complex SWR-C; by altering the tendency of the protein to be removed by INO80-C; and by causing a general destabilisation of the protein’s association with chromatin. These findings underline the importance of normal chromatin residency for Htz1, as these mutations that affect chromatin occupancy are functionally defective.

## Methods

### Yeast strains, plasmids and molecular cloning

Strains of *S. cerevisiae* and plasmids used in this study are listed in Tables S1 and S2. Gene epitope-tagging and deletion were performed using standard techniques [40].

### Generation of a library of randomly mutated *HTZ1* alleles

The *HTZ1* coding sequence and a small amount of flanking sequence were amplified by error-prone (EP) PCR for 20 cycles using SAWADY Taq DNA polymerase (Peqlab) in the presence of 7 mM MgCl_2_, 0.5 mM MnCl_2_ and a 1:5:1:5 ratio of dATP:dCTP:dGTP:dTTP. 10 separate EP-PCR reactions were performed and pooled to minimise jackpot effects. EP-PCR products were cloned into pCM433 [35] to create a library of plasmids containing randomly mutagenised *htz1* alleles.

### Screening the library of randomly mutated *HTZ1* alleles in *S. cerevisiae*

Yeast strain CMY307, deleted for *ASF1* and containing wild-type (WT) *3HA-HTZ1* under the control of the *GAL1* promoter, was transformed with the randomly mutagenised library. Transformants were selected on synthetic medium lacking uracil and containing 2% galactose. 1,453 separate URA+ transformants were gridded into 96-well format from the original transformation and screened for growth under conditions where WT *HTZ1* was shut off by glucose repression (established by western blotting for HA-Htz1). Controls (no yeast cells; empty vector and WT *HTZ1*) were included on each plate and growth was scored (0-4) relative to the empty vector (pRS416) and WT plasmid (pCM433) respectively. Clones that scored 0-2 in the first round of screening were re-gridded into new positions to rule out plate position effects and re-screened; those that scored 0-2 in the second round of screening were selected as unable to provide the function of Htz1. 149 clones were selected after the second round of screening; the corresponding plasmids were rescued from yeast, passed through *E. coli* and sequenced. All yeast strains and plasmids are described in Tables S1 and S2.

### Spot test assays

Saturated cultures were serially diluted 1:5 before spotting onto SD-ura agar plates containing either 2% (v/v) formamide, 5 mM caffeine, 75 mM hydroxyurea, 0.0075% (v/v) MMS, 15 µg/ml benomyl, or without any drug. Plates were incubated at 30°C for 48 hours.

### Cell fractionation and chromatin stability assay

Log phase cells were harvested, and fractionated as described in [35] to obtain whole cell lysates, and separate cytoplasmic and nuclear material. In cell fractionation assays, a Tris buffer containing 1% Triton and 400 mM NaCl was used to lyse nuclei and obtain the insoluble chromatin pellet. In the chromatin stability assay, nuclei were lysed with a 100 mM NaCl 1% Triton Tris buffer, and then divided into equal aliquots before washing with 100, 200, 300 or 400 mM NaCl 1% Triton Tris buffers. Proteins within soluble cell fractions and insoluble chromatin pellets were analysed by Western blotting.

### Chromatin Immunoprecipitation (ChIP)

ChIP was performed as described in [35]. Recovered ChIP DNA and cell lysate (input) DNA were purified using the QIAquick PCR Purification Kit (Qiagen) and analysed by Q-PCR in triplicate. Primer pairs (sequences available upon request) were validated for equivalent linear amplification. Input-normalised enrichment values for each target were calculated relative to a negative control region within the silent *HMR* locus.

### Co-immunoprecipitation (co-IP) assay

Log phase cells were harvested, resuspended in co-IP buffer [22] and lysed with 0.5 mm glass beads (BioSpec) in a FastPrep-24 (MP Biomedicals) cell homogeniser.

Cell debris was then pelleted by centrifugation and the soluble supernatant was incubated with monoclonal anti-HA agarose conjugated beads (Sigma A2095) overnight at 4°C. Beads were washed three times using co-IP buffer and then eluted with 2X Laemmli SDS-PAGE loading buffer. Whole cell lysate (input) and immunoprecipitated samples (IP) were analysed by Western blotting.

### Western blotting and quantification

Protein samples were analysed by SDS-PAGE separation and transfer onto nitrocellulose membranes. Membranes were probed using primary antibodies specific for HA (12CA5; Roche), histone H4 (Abcam ab10158), glucose-6-phosphate dehydrogenase (G6PDH; Sigma A9531), Flag (Sigma F3165) and Chz1 [36]. These were detected and the signal quantified using IRDye-conjugated secondary antibodies and by scanning membranes on the Odyssey Infrared Imaging System (LI-COR Biosciences). Antibodies were used at concentrations at which the detected signal varied linearly with sample protein concentration. The integrated intensity values for specific bands were measured and loading-normalised values were produced for each sample. These values were then log transformed prior to analysis with a two-tailed paired *t*-test to assess statistical significance.

### Structural modelling

In order to model the effect of mutations, the crystal structure of the mouse H2A.Z-containing nucleosome (PDB code 1F66) was used. Hydrogen atoms were added using the “Reduce” software [41]. Mutations were made using the side chain mutator tool in KiNG [42] to simulate mutations. All low energy side chain conformations (rotamers) were considered and the all-atom contacts calculated [38] to visualise van der Waals contacts and hydrogen bonds. In each case the rotamer with the fewest van der Waals overlaps was considered as the most likely, and this conformation is shown in Figure 5.

## Supporting Information

Figure S1 **Mutant Htz1 proteins interact with Chz1.** Anti-HA antibodies were used to immunoprecipitate cell lysates from strains expressing either HA-tagged WT, mutant, or no Htz1 (htz1∆). Input and anti-HA IP samples were analysed by anti-HA and anti-Chz1 immunoblotting, with an example blot shown on the left. The position of the antibody light chain is indicated (*). Levels of co-immunoprecipitated Chz1 for each strain were normalised to the amount of immunoprecipitated HA-Htz1, expressed relative to WT, and averages are depicted in the graph (right; n = 3). Error bars indicate standard error of the mean. Asterisks indicate the results of two-tailed paired *t*-tests between the indicated strains, where * = *P* < 0.01, ** = *P* < 0.005.
(TIF)

Figure S2 **Mutant Htz1 proteins have reduced interaction with the SWR-C subunit, Arp6.** Anti-HA antibodies were used to immunoprecipitate cell lysates from strains expressing FLAG-tagged Arp6 and either HA-tagged WT, mutant, or no Htz1 (*htz1∆*). Input and anti-HA IP samples were analysed by anti-HA and anti-FLAG immunoblotting, with an example blot shown on the left. The positions of the antibody light and heavy chains are indicated in the anti-HA and anti-FLAG Western blots respectively (*). Levels of co-immunoprecipitated Arp6-FLAG for each strain were normalised to the amount of immunoprecipitated HA-Htz1, expressed relative to WT, and averages are depicted in the graph (right; n = 4). Error bars indicate standard error of the mean. Asterisks indicate the results of two-tailed paired *t*-tests between the indicated strains, where * = *P* < 0.05, ** = *P* < 0.001.
(TIF)

Figure S3 **Deletion of the INO80 subunit, Arp5, increases the level of mutant Htz1 in chromatin.** **[A]** Representative Western blots of sub-cellular fractions generated from WT and *arp5∆*cells, labelled as in Figure 2B. Chromatin HA-Htz1 protein levels normalised to H4, expressed relative to WT and averaged are shown on the right (n = 3). **[B]** Representative Western blot of chromatin generated from single *htz1* mutants and *arp5∆* double mutants. Quantification of chromatin HA-Htz1 protein levels as in **[A]** but where each double mutant is compared to the corresponding single HA-Htz1 mutant, is shown on the right (n = 3). Error bars indicate standard error of the mean. The asterisk indicates the result of a two tailed paired *t*-test between the indicated strains, where * = *P* < 0.005.
(TIF)

Table S1 ***S. cerevisiae* strains.**
(DOC)

Table S2 **Plasmid constructs.**
(DOC)
