# Supplementary figures and images for: Mutations in Non-Acid Patch Residues Disrupt H2A.Z’s Association with Chromatin through Multiple Mechanisms
Source: PLoS One. 2013 Oct 1;8(10):e76394. doi: 10.1371/journal.pone.0076394 (PMC3788105; doi:10.1371/journal.pone.0076394)

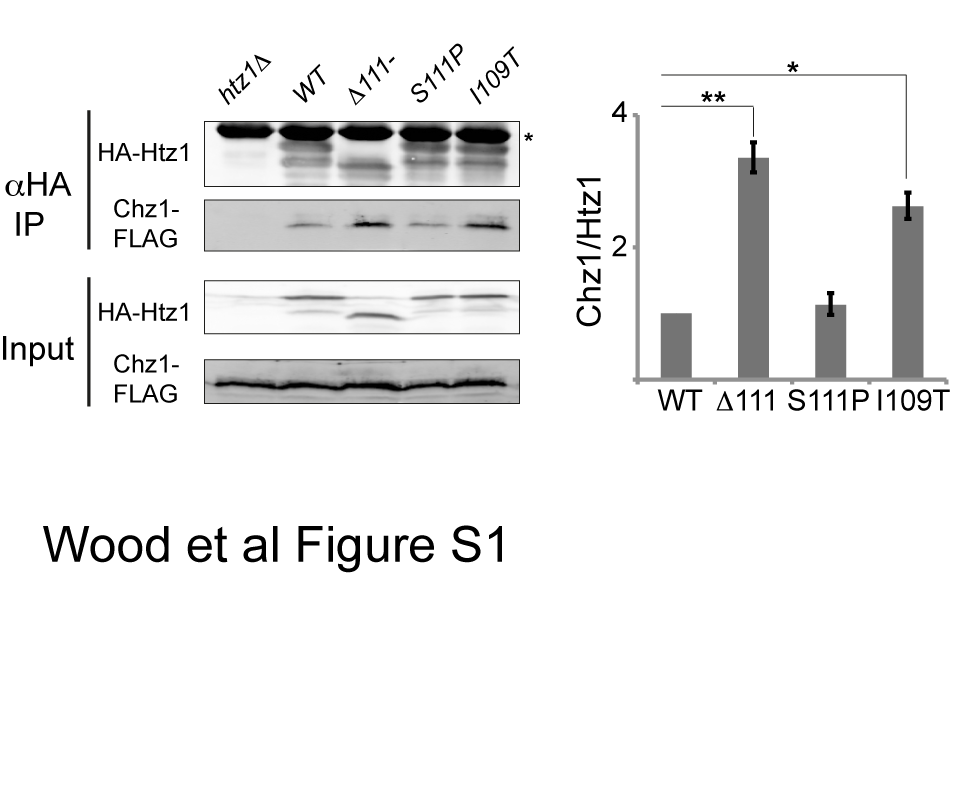

Supplement: Figure S1 — Mutant Htz1 proteins interact with Chz1. Anti-HA antibodies were used to immunoprecipitate cell lysates from strains expressing either HA-tagged WT, mutant, or no Htz1 (htz1∆). Input and anti-HA IP samples were analysed by anti-HA and anti-Chz1 immunoblotting, with an example blot shown on the left. The position of the antibody light chain is indicated (*). Levels of co-immunoprecipitated Chz1 for each strain were normalised to the amount of immunoprecipitated HA-Htz1, expressed relative to WT, and averages are depicted in the graph (right; n = 3). Error bars indicate standard error of the mean. Asterisks indicate the results of two-tailed paired t-tests between the indicated strains, where * = P < 0.01, ** = P < 0.005. (TIF) [file pone.0076394.s001.tif]

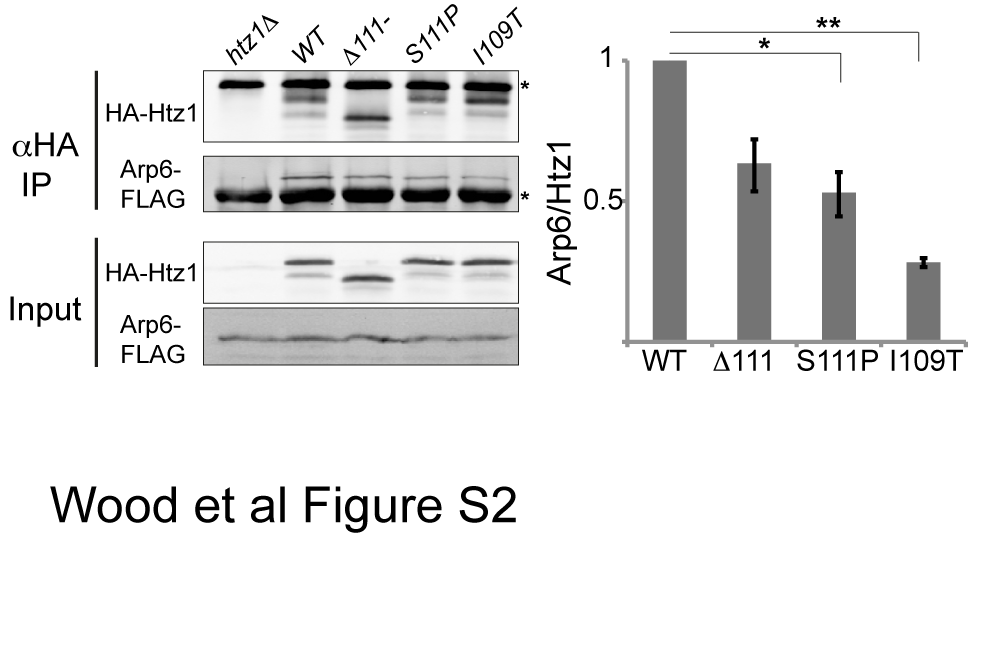

Supplement: Figure S2 — Mutant Htz1 proteins have reduced interaction with the SWR-C subunit, Arp6. Anti-HA antibodies were used to immunoprecipitate cell lysates from strains expressing FLAG-tagged Arp6 and either HA-tagged WT, mutant, or no Htz1 (htz1∆). Input and anti-HA IP samples were analysed by anti-HA and anti-FLAG immunoblotting, with an example blot shown on the left. The positions of the antibody light and heavy chains are indicated in the anti-HA and anti-FLAG Western blots respectively (*). Levels of co-immunoprecipitated Arp6-FLAG for each strain were normalised to the amount of immunoprecipitated HA-Htz1, expressed relative to WT, and averages are depicted in the graph (right; n = 4). Error bars indicate standard error of the mean. Asterisks indicate the results of two-tailed paired t-tests between the indicated strains, where * = P < 0.05, ** = P < 0.001. (TIF) [file pone.0076394.s002.tif]

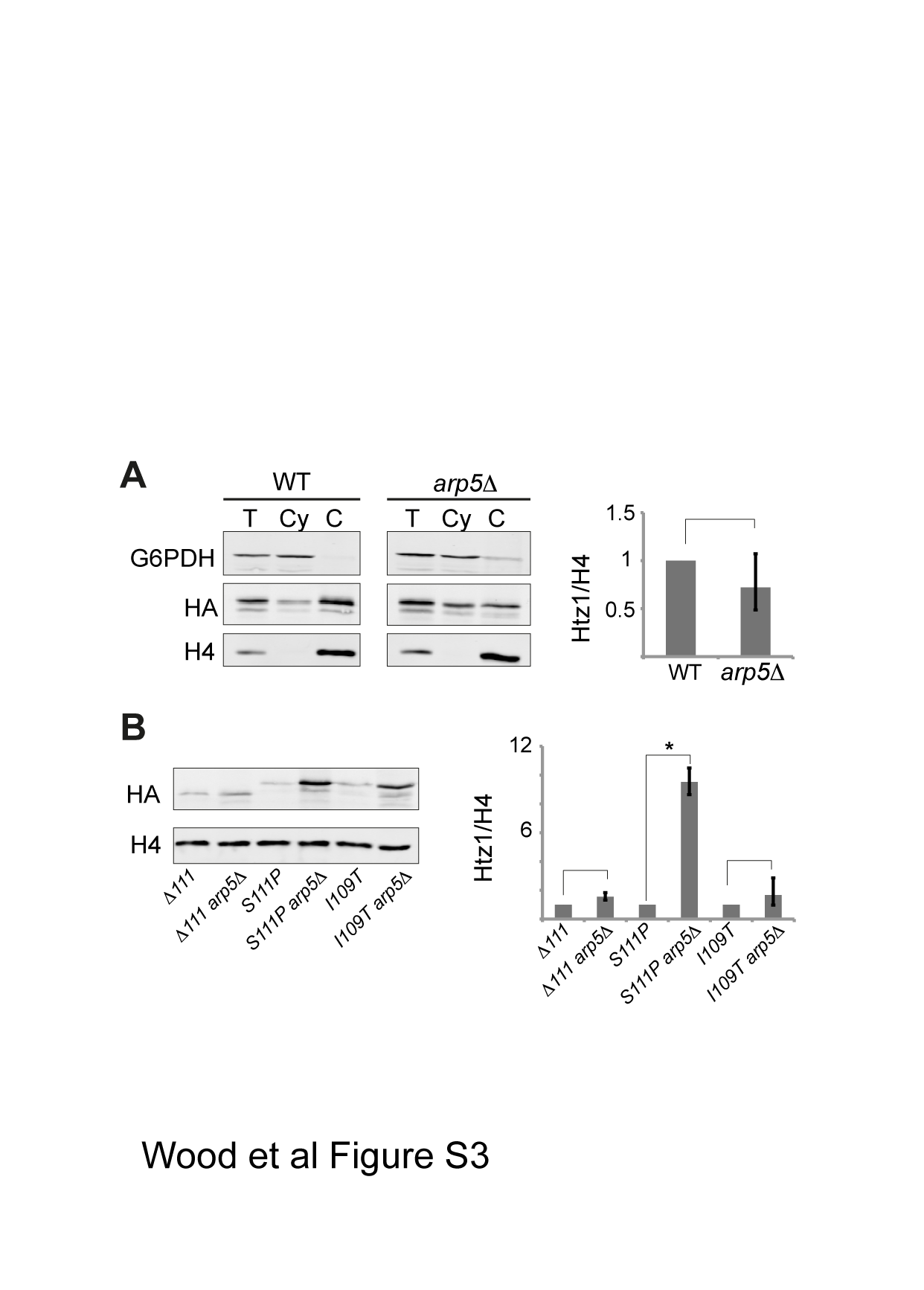

Supplement: Figure S3 — Deletion of the INO80 subunit, Arp5, increases the level of mutant Htz1 in chromatin. [A] Representative Western blots of sub-cellular fractions generated from WT and arp5∆cells, labelled as in Figure 2B. Chromatin HA-Htz1 protein levels normalised to H4, expressed relative to WT and averaged are shown on the right (n = 3). [B] Representative Western blot of chromatin generated from single htz1 mutants and arp5∆ double mutants. Quantification of chromatin HA-Htz1 protein levels as in [A] but where each double mutant is compared to the corresponding single HA-Htz1 mutant, is shown on the right (n = 3). Error bars indicate standard error of the mean. The asterisk indicates the result of a two tailed paired t-test between the indicated strains, where * = P < 0.005. (TIF) [file pone.0076394.s003.tif]
