# Supplementary material for: Mutations in Non-Acid Patch Residues Disrupt H2A.Z’s Association with Chromatin through Multiple Mechanisms
Source: PLoS One. 2013 Oct 1;8(10):e76394. doi: 10.1371/journal.pone.0076394 (PMC3788105; doi:10.1371/journal.pone.0076394)
Supplement: Table S1 — S. cerevisiae strains. (DOC) [file pone.0076394.s004.doc]

**Wood et al**

**Supplemental Table** **1** *S. cerevisiae* strains.

| Name | Genotype | Reference |
| --- | --- | --- |
| CMY307 | *MATa, ade2-1, can1-100, his3-11, leu2-3, 112, trp1-1, ura3-1, P-GAL1-HAHTZ1-KanMX, asf1*Δ*::His3MX6* | This study |
| CMY665 | YDS2, *bar1Δ::His3MX6, htz1Δ::TRP1* [pRS416] | This study |
| CMY666 | YDS2, *bar1Δ::His3MX6, htz1Δ::TRP1* [pCM433] | This study |
| CMY667 | YDS2, *bar1Δ::His3MX6, htz1Δ::TRP1* [pZRM7] | This study |
| CMY668 | YDS2, *bar1Δ::His3MX6, htz1Δ::TRP1* [pZRM19] | This study |
| CMY669 | YDS2, *bar1Δ::His3MX6, htz1Δ::TRP1* [pZRM15] | This study |
| CMY394 | YDS2, *bar1Δ::His3MX6, htz1Δ::TRP1* [pCM305] | This study |
| CMY425 | YDS2, *bar1Δ::His3MX6, htz1Δ::TRP1* [pCM507] | This study |
| CMY391 | YDS2, *bar1Δ::His3MX6, htz1Δ::TRP1* [pCM482] | This study |
| CMY670 | YDS2, *bar1Δ::His3MX6, htz1Δ::TRP1* [pCM628] | This study |
| CMY863 | YDS2, *bar1Δ::His3MX6, htz1Δ::TRP1* *SWC2-FLAG::KanMX,* [pCM305] | This study |
| CMY864 | YDS2, *bar1Δ::His3MX6, htz1Δ::TRP1* *SWC2-FLAG::KanMX,* [pCM482] | This study |
| CMY865 | YDS2, *bar1Δ::His3MX6, htz1Δ::TRP1* *SWC2-FLAG::KanMX* [pCM507] | This study |
| CMY866 | YDS2, *bar1Δ::His3MX6, htz1Δ::TRP1* *SWC2-FLAG::KanMX* [pCM628] | This study |
| CMY867 | YDS2, *bar1Δ::His3MX6, htz1Δ::TRP1* *SWC2-FLAG::KanMX* [pRS416] | This study |
| CMY676 | YDS2, *bar1Δ::His3MX6, htz1Δ::TRP1* *swr1Δ::KanMX* [pCM628] | This study |
| CMY677 | YDS2, *bar1Δ::His3MX6, htz1Δ::TRP1* *swr1Δ::KanMX* [pCM305] | This study |
| CMY678 | YDS2, *bar1Δ::His3MX6, htz1Δ::TRP1* *swr1Δ::KanMX* [pCM482] | This study |
| CMY679 | YDS2, *bar1Δ::His3MX6, htz1Δ::TRP1* *swr1Δ::KanMX* [pCM507] | This study |
| CMY750 | YDS2, *htz1Δ::TRP1* [pCM303] | This study |
| CMY951 | YDS2, *htz1Δ::TRP1* [pCM638] | This study |
| CMY952 | YDS2, *htz1Δ::TRP1* [pCM639] | This study |
| CMY953 | YDS2, *htz1Δ::TRP1* [pCM640] | This study |
| CMY751 | YDS2, *htz1Δ::TRP1, arp8Δ::KanMX* [pCM303] | This study |
| CMY752 | YDS2, *htz1Δ::TRP1, arp8Δ::KanMX* [pCM638] | This study |
| CMY753 | YDS2, *htz1Δ::TRP1, arp8Δ::KanMX* [pCM639] | This study |
| CMY754 | YDS2, *htz1Δ::TRP1, arp8Δ::KanMX* [pCM640] | This study |
| CMY1023 | YDS2, *bar1Δ::His3MX6, htz1Δ::TRP1* *ARP6-FLAG::KanMX,* [pRS416] | This study |
| CMY1024 | YDS2, *bar1Δ::His3MX6, htz1Δ::TRP1* *ARP6-FLAG::KanMX,* [pCM305] | This study |
| CMY1025 | YDS2, *bar1Δ::His3MX6, htz1Δ::TRP1* *ARP6-FLAG::KanMX,* [pCM507] | This study |
| CMY1026 | YDS2, *bar1Δ::His3MX6, htz1Δ::TRP1* *ARP6-FLAG::KanMX,* [pCM482] | This study |
| CMY1027 | YDS2, *bar1Δ::His3MX6, htz1Δ::TRP1* *ARP6-FLAG::KanMX,* [pCM628] | This study |
| CMY1034 | YDS2, *bar1Δ::His3MX6, htz1Δ::TRP1* *arp5Δ::KanMX* [pCM303] | This study |
| CMY1035 | YDS2, *bar1Δ::His3MX6, htz1Δ::TRP1* *arp5Δ::KanMX* [pCM639] | This study |
| CMY1036 | YDS2, *bar1Δ::His3MX6, htz1Δ::TRP1* *arp5Δ::KanMX* [pCM638] | This study |
| CMY1037 | YDS2, *bar1Δ::His3MX6, htz1Δ::TRP1* *arp5Δ::KanMX* [pCM640] | This study |
| CMY1043 | YDS2, *bar1Δ::His3MX6, htz1Δ::TRP1* [pCM303] | This study |
| CMY1044 | YDS2, *bar1Δ::His3MX6, htz1Δ::TRP1* [pCM639] | This study |
| CMY1045 | YDS2, *bar1Δ::His3MX6, htz1Δ::TRP1* [pCM638] | This study |
| CMY1046 | YDS2, *bar1Δ::His3MX6, htz1Δ::TRP1* [pCM640] | This study |
