# Supplementary material for: Mutations in Non-Acid Patch Residues Disrupt H2A.Z’s Association with Chromatin through Multiple Mechanisms
Source: PLoS One. 2013 Oct 1;8(10):e76394. doi: 10.1371/journal.pone.0076394 (PMC3788105; doi:10.1371/journal.pone.0076394)
Supplement: Table S2 — Plasmid constructs. (DOC) [file pone.0076394.s005.doc]

**Wood et al**

**Supplemental Table 2 Plasmid constructs.**

| **Name** | **Description** | **Reference** |
| --- | --- | --- |
| pRS416 | *CEN6-ARS4 URA3* | [S1] |
| pCM305 | *CEN6-ARS4 URA3 3HA-HTZ1* | [23] |
| pCM433 | *CEN6-ARS4 URA3 HTZ1* | [35] |
| pZRM7 | *CEN6-ARS4 URA3 htz1Δ111-133* |  |
| pZRM19 | *CEN6-ARS4 URA3 htz1S111P* | This study |
| pZRM15 | *CEN6-ARS4 URA3 htz1I109T* | This study |
| pCM507 | *CEN6-ARS4 URA3 3HA-htz1Δ111-133* | This study |
| pCM482 | *CEN6-ARS4 URA3 3HA-htz1S111P* | This study |
| pCM628 | *CEN6-ARS4 URA3 3HA-htz1I109T* | This study |
| pRS415 | *CEN6-ARS4 LEU2* | [S1] |
| pCM303 | *CEN6-ARS4 LEU2 3HA-HTZ1* | This study |
| pCM638 | *CEN6-ARS4 LEU2 3HA-htz1S111P* | This study |
| pCM639 | *CEN6-ARS4 LEU2 3HA-htz1Δ111-133* | This study |
| pCM640 | *CEN6-ARS4 LEU2 3HA-htz1I109T* | This study |

Supplemental references

S1. Sikorski RS, Hieter P (1989) A system of shuttle vectors and yeast host strains designed for efficient manipulation of DNA in Saccharomyces cerevisiae. Genetics 122: 19–27.
